# Barbie-Cueing Weight Perception

**DOI:** 10.1177/2041669519850590

**Published:** 2019-05-10

**Authors:** Elizabeth J. Saccone, Philippe A. Chouinard

**Affiliations:** Department of Psychology and Counselling, School of Psychology and Public Health, La Trobe University, Melbourne, Victoria, Australia

**Keywords:** perception/action, top-down perception, weight illusions, weight perception

## Abstract

It was previously reported that Barbie feels heavier than Ken when both dolls are matched for mass. However, we felt it was unclear from this earlier report if the effects went beyond a typical size-weight illusion. By providing better controls, we conclude more confidently that doll features other than size influence weight perception. Specifically, conceptual knowledge, in the form of culturally reinforced biases, seems to affect how we perceive their weight.

Through imaginative play, children pretend that toys are machines, animals, and people from the real world. In making these toys, toy companies sometimes exaggerate particular features associated with their real-world counterparts. Consider Barbie and Ken dolls (Mattel Inc., El Segundo, CA, USA). Ken portrays a youthful, masculine man with a lean, muscular physique, whereas Barbie emulates more feminine qualities with her smaller stature and overly exaggerated, unrealistic figure. In a truly unique study, Dijker (2008) used dolls to investigate how exaggerated features, and any (implicit or explicit) associations we might have about them, can influence their expected and perceived heaviness. He hypothesised that people would expect Ken-like dolls to be heavier than Barbie-like dolls, given our culturally reinforced biases from childhood, and that these expectations would also affect perceived weight.

Dijker (2008) reengineered the dolls to have equal mass to determine if people’s perception of their weight would follow a size-weight illusion (SWI)—a well-documented phenomenon in which the smaller of two objects of equal mass feels heavier (Charpentier, 1891; for reviews, see Dijker, 2014; Saccone & Chouinard, 2019). One theory, the sensorimotor mismatch theory, proposes that the apparent weight differences are driven by a mismatch between expected and experienced weight (Dijker, 2014). More precisely, people expect the smaller object to be lighter and therefore apply less force than is required to lift it. Additional force is needed, causing the object to feel heavier. Conversely, the larger object is lifted with excessive force, and applying corrective forces to stabilise it in the air causes it to feel lighter.

Because Dijker (2008) was interested in the influence of culturally reinforced biases, he created a cohort of uncanny dolls—each with the same mass but with an exaggerated quality. Specifically, the dolls emphasised cuteness, youth, old age, masculinity, femininity, different races, or some combination of these. Participants rated the dolls’ expected (pre-lift) weight and perceived (post-lift) weight. As hypothesised, culturally reinforced biases seemed to affect both measurements. Participants expected dolls emulating strength and masculinity to weigh more, yet perceived them to weigh less. Participants in a control experiment lifted a series of cans that varied in volume but weighed the same as the dolls. Participants reported an SWI; smaller cans felt heavier. Still, this illusion seemed weaker than that found with the dolls. Taken together, Dijker concluded that culturally reinforced biases influenced the dolls’ perceived weight beyond a simple SWI.

However, we felt that there were some shortcomings with the design. Dijker (2008) did not report the volume of the dolls nor did he match the cans’ volumes to the dolls. In our view, it is imperative that control objects match the dolls in volume as well as weight to confidently conclude that the dolls elicit a stronger weight illusion than a typical SWI. Thus, Experiment 1 aimed to do precisely that, using 31 right-handed adults from the La Trobe University community (16 females, 15 males; *M*_age_ = 23.84 years, *SD*_age_ = 4.68). All participants in this study gave written informed consent to the procedures, which were approved by the University’s ethics committee.

Stimuli were two sets of objects (i.e., dolls and control objects), matched in weight, volume, height, and colour (Figure 1(a) and (b)). The dolls were Ken (*Denim Blues* model) and Barbie (*Love That Lace* model) from Mattel’s *2016 Fashionistas* series, which graced the cover of Time magazine (not as *Person of the Year*, which went to President Trump instead). The dolls underwent *body sculpting* in our workshop until they both weighed 122 g. We drilled holes in Ken to reduce his weight and implanted lead in Barbie’s back to increase hers (Figure 1(c) and (d)).

**Figure 1.**
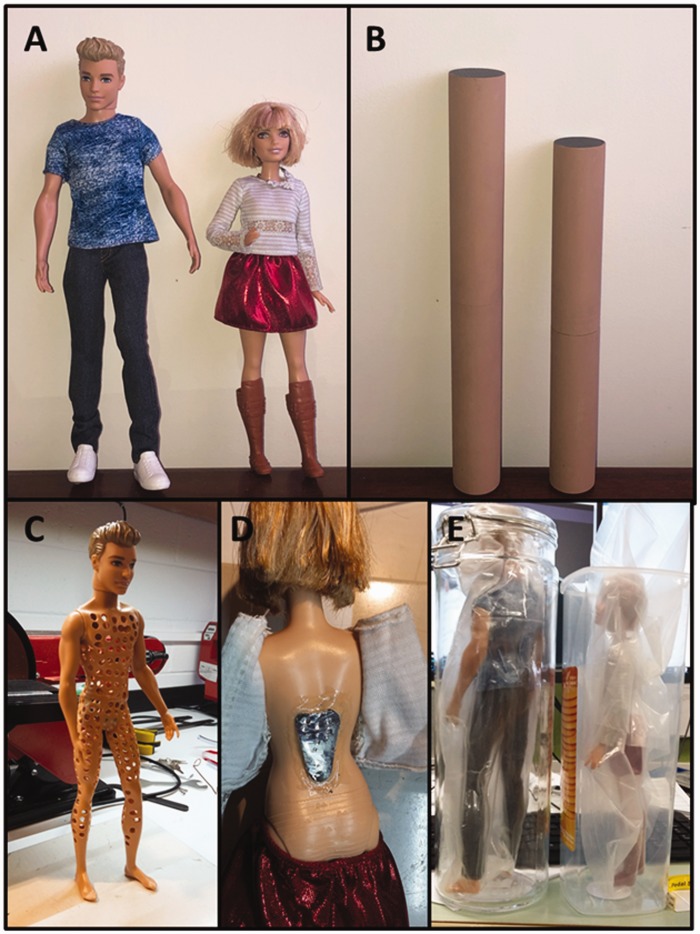
The Ken and Barbie dolls (a) and cylinders (b) used in the experiments. We drilled holes in Ken to reduce his weight (c) and added lead to Barbie to increase hers (d). Ken and Barbie’s volumes were determined by method of water displacement (e). The dolls did not experience pain or drowning and tolerated their treatments well given their inanimate disposition.

Following these treatments, we gave the dolls a bath to determine the amount of water displaced after submersion (Figure 1(e)). These values were taken as their volumes. Using this information, we created the control objects—3D-printed cylinders—that matched the dolls in both volume (Ken: 328.25 cm^3^, Barbie: 246.29 cm^3^) and height (Ken: 31.2 cm, Barbie: 26.5 cm). The cylinders were painted a similar colour to the dolls’ skin, because colour can influence perceived weight (Walker, Francis, & Walker, 2010). Finally, we inserted lead pellets in the centre of the cylinders, held in place by foam, so that they also weighed 122 g.

In Experiment 1, participants closed their eyes and one stimulus pair (dolls or cylinders) was placed on the table in front of them. Participants then opened their eyes, hefted one stimulus at a time, and provided heaviness ratings using an absolute magnitude estimation procedure described elsewhere (Buckingham & Goodale, 2013). They used a different hand for each stimulus and then lifted each of them with the opposite hands. This procedure was repeated for the second stimulus pair. The order in which the pairs were presented, and the starting hand used to heft the stimuli, was counterbalanced across participants.

Participants’ ratings were standardised into *Z*-scores by subtracting each value from their mean, divided by the standard deviation. A 2 (Object: dolls, cylinders) × 2 (Size: small, large) repeated measures analysis of variance was performed on the standardised ratings (Figure 2(a)). There was a main effect of Object, *F*(1, 30) = 14.75, *p* < .001, $\eta_{p}^{2}$ = .330, reflecting higher estimates for the cylinders than dolls. There was also a main effect of Size, *F*(1, 30) = 35.34, *p* < .001, $\eta_{p}^{2}$ = .541, with the small doll/cylinder rated as heavier than the large doll/cylinder. The interaction was significant, *F*(1, 30) = 4.93, *p* = .034, $\eta_{p}^{2}$ = .141, reflecting a greater difference between Barbie and Ken, *t*(30) = 6.22, *p* < .001, Cohen’s *d* = 1.12, than the cylinders, *t*(30) = 2.93, *p* = .006, Cohen’s *d* = .53.

**Figure 2.**
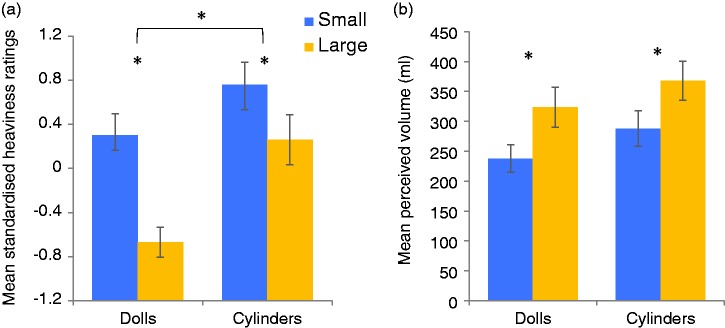
Mean standardised heaviness ratings (a; Experiment 1) and perceived volume as measured with the water task (b; Experiment 2) with standard errors for the small (blue) and large (yellow) dolls and cylinders. Asterisks (*) denote significant differences after correcting for multiple comparisons (*p* < .05).

Although the pairs were matched in physical volume, the greater perceived weight difference for Barbie and Ken could potentially be explained by a greater difference in *perceived* volume for the dolls, given that object shape and structure can influence perceived volume (Raghubir & Krishna, 1999). Thus, in Experiment 2, 16 new right-handers (8 females, 8 males, *M*_age_ = 21.56 years, *SD*_age_ = 1.27) indicated the perceived volume of each doll/cylinder by pouring a representative amount of water from a large, transparent container (capacity: 1.8 L; approximately 8.5 cm × 8.5 cm × 26 cm) into a smaller, transparent container (capacity: 0.9 L; approximately 9 cm × 9 cm × 12 cm). They were asked to pour the amount of liquid they felt would fill each stimulus if it were hollow. Stimuli were presented as pairs (with appropriate counterbalancing as in Experiment 1) and participants performed two trials for each pair. They did not touch the objects.

Measuring the amount of water (millilitres) poured to represent each stimulus demonstrated that participants did not perceive greater differences in volume between the dolls than the cylinders. A 2 (Object) × 2 (Size) repeated measures analysis of variance (see Figure 2(b)) demonstrated a main effect of Size, *F*(1, 15) = 46.08, *p* < .001, $\eta_{p}^{2}$ = .754, with greater volumes of water assigned for the large objects, but no main effect of Object, *F*(1, 15) = 2.78, *p* = .12, $\eta_{p}^{2}$ = .156, or interaction, *F*(1, 15) = 0.10, *p* = .76, $\eta_{p}^{2}$ = .007. Participants also provided magnitude estimates of volume for each stimulus, consistent with the procedures in Experiment 1, which produced the same pattern of data—Size, *F*(1, 15) = 165.54, *p* < .001, $\eta_{p}^{2}$ = .917; Object, *F*(1, 15) = 1.54, *p* = .23, $\eta_{p}^{2}$ = .093, Size × Object, *F*(1, 15) = 1.64, *p* = .22, $\eta_{p}^{2}$ = .099. These two approaches to measuring perceptual volume correlated with each other, *r*(62) = .44, *p* < .001.

These results are informative. We can now say with more confidence that dolls can influence weight perception beyond a simple SWI, and that this difference is not due to either physical or perceived differences in volume. Conceptual knowledge, in the form of culturally reinforced biases, seems to affect how we perceive their weight. This has important theoretical implications as it suggests that weight perception can be influenced by a top-down mechanism. Whether or not the features represented by dolls influence perception via variations in lifting behaviour remains under debate (see Dijker, 2008, for his original proposals). The findings also demonstrate how cultural biases can permeate even basic perceptual processing, including our conscious experience of the weight of objects around us.
